# Physical evaluation of a new pulp capping material developed from portland cement

**DOI:** 10.4317/jced.52748

**Published:** 2016-07-01

**Authors:** Ahmed Negm, Ehab Hassanien, Ashraf Abu-Seida, Mohamed Nagy

**Affiliations:** 1 MSc. Assistant lecturer october 6 university, 3 mohamed kamel morsy dokki, Giza Egypt; 2 PhD, Professor of Endodontics. Department of Endodontics. Faculty of Dentistry. Ain Shams University. Cairo. Egypt; 3 PhD, Professor. Department of Surgery, Anesthesiology and Radiology, Faculty of Veterinary Medicine, Cairo University, Giza-Egypt; 4 PhD, Lecturer of Endodontics. Department of Endodontics. Faculty of Dentistry. Ain Shams University. Cairo. Egypt

## Abstract

**Background:**

This study examined the effects of addition of 10% and 25% by weight calcium hydroxide on the physicochemical properties of Portland cement associated with 20% bismuth oxide in order to develop a new pulp capping material.

**Material and Methods:**

The solubility, pH value, setting time, compressive strength, and push out bond strength of modified Portland were evaluated and compared to those of mineral trioxide aggregate (MTA) and Portland cement containing 20% bismuth oxide.

**Results:**

The statistical analysis was performed with ANOVA and Duncan’s post-hoc test. The results show that the strength properties and push out bond strength of Portland cement were adversely affected by addition of calcium hydroxide especially with a ratio of 25 wt%, however, the setting time and pH were not affected. MTA showed a statistically significant lower setting time than other cements (*P*≤0.001). Portland cement with bismuth oxide and Port Cal I showed a statistically significant higher Push out Bond strength than MTA and Port Cal II (*P*=0.001).

**Conclusions:**

Taking the setting time, push out bond strength and pH value into account, addition of 10 wt% calcium hydroxide to Portland cement associated with 20% bismuth oxide produces a new pulp capping material with acceptable physical and adhesive properties. Further studies are recommended to test this cement biologically as a new pulp capping material.

** Key words:**Calcium hydroxide, MTA, Portland cement, setting time, solubility, strength.

## Introduction

Pulp capping is defined as the treatment of exposed vital pulp by the application of capping materials to induce the dentinogenic potential of pulp cells (1). The choice of pulp capping material greatly influences the success of vital pulp therapy. Mineral trioxide aggregate (MTA) was introduced by Torabinejad *et al.* (2) in early nineties. It has been recommended as a material for pulp capping, root canal filling, perforation repair, apexification, apical barriers and revascularization (3,4).

MTA is a mixture of Portland cement (75%), bismuth oxide (20%), gypsum (5%) and trace amounts of SiO2, CaO, MgO, K2SO4 and Na2SO4.

The four major compounds in Portland cement (PC) are: dicalcium silicate, tricalcium silicate, tricalcium aluminate, and tetracalcium aluminoferrite. The silicates are the responsible ingredients for the strength of hydrated cement paste.

In the last few years, many researchers had focused on the similarities between Portland cement and MTA in order to search for a cheaper substitute for the expensive MTA. The major ingredients of MTA as published by Koh *et al.* (5) matched the primary ingredients of PC given by the Portland cement association.

Funteas *et al.* (6) found a similar composition and antimicrobial action when comparing MTA (ProRoot) and Portland cement, except absence of bismuth oxide in Portland cement, which is the radiopacifier of ProRoot. Only *E. coli* was not inhibited by MTA and Portland Cement. In addition, Portland cement has similar or better sealing ability of furcal perforations than MTA (7).

Calcium hydroxide and calcium hydroxide compounds are the gold standard in human teeth, against which new materials should be evaluated (8). However, several disadvantages have been reported with the use of calcium hydroxide material such as presence of tunnels in dentin barrier, extensive dentin formation obliterating the pulp chamber, lack of adhesion and degradation after acid etching and high solubility in oral fluids, it has antibacterial effect (9). It produced calcified degenerative zone that had an important effect on the reparative process of pulp tissue after pulpotomy (10).

Pulps capped with ProRoot white MTA and white Portland cement exhibited thicker reparative hard tissue deposition over the exposed pulps than those capped with Ca(OH)2 (11). However, teeth capped with calcium hydroxide had statistically favorable outcomes in hard tissue formation and pulp inflammation (12).

The aim of present part of the study was to evaluate the physicochemical properties of Portland cement associated with bismuth oxide and calcium hydroxide in an attempt to develop a new pulp capping material.

## Material and Methods

-Formation of the new material:

This study was approved by the Ethics Committee of Faculty of Dentistry, Ainshams University (2013/03END). Bismuth oxide (Loba Chemie, India) was incorporated into Portland cement (ASEC Helwan cement, Egypt) in the ratio of 20% by weight. The calcium hydroxide powder (ANALAR, Oxford laboratory. Mumbai, India) was then mixed with Portland cement in two different ratios, 10 wt% and 25 wt%.

The ingredients of the powder were blended together in a vibratory mixer for one hour. The resultant mixtures were mixed with distilled water and the newly formed cements were designated Portland Cal I and Portland Cal II (Port Cal I and Port Cal II).

-Sample grouping:

Four main groups were used in this study including; group 1, MTA (Endocem Maruchi, Korea); Group 2, Portland cement + bis-muth oxide; group 3, Port Cal I and group 4, Port Cal II. Eight samples of cements were applied in each test.

-Determination of powder/water ratio of the experimental cements:

The powder/water ratio of 1/1 by weight was tried first. The powder and water were weighed out on a glass slab at room temperature. The powder was then divided into four equal portions on the glass slab. Mixing was started by incorporation of the first portion of the powder into the water. The process was continued to gain the desired consistency. The ultimate amount of powder that can be incorporated in the water to attain a homogenous consistency was used. Thus, the amount of powder to water needed for mixing was determined and recorded as (3:1).

-Standardization of the setting time of the cements:

The experimental cements were mixed according to the predetermined powder/water ratio. The soft mixes were immediately poured into steel moulds (10mm diameter and 4mm depth) and kept in an oven at 37oC and relative humidity. Setting time was tested every 5 minutes for each material using a Gilmore needle weighing 300gm with a diameter of 1mm. As soon as the needle caused a superficial indentation on the surface, testing of the setting time was done every one minute. Each specimen of a series was subject to the needle one time only to avoid any interference by the surface irregularities formed by previous testing.

The initial setting time for each specimen was recorded when the needle caused a superficial indentation on the surface (less than 1mm depth). The time at which the needle was no longer able to produce any surface mark was recorded as the final setting time. Setting time of the Portland cement was similarly tested.

-Testing of pH of the cements:

Each cement was mixed and filled into polypropylene test tubes having a diameter of 7.75mm and a height of 1.5mm (13). Immediately after mixing and filling, the test tube was carefully lowered into a plastic cup containing 4ml isotonic saline. The test tube, containing the mix, was kept in the first cup for 1 minute then moved to the second cup and kept in it for 4 minutes. It was then immersed in the third cup for 10 minutes, followed by immersion periods of 15 minutes each in every cup after the third one. This process was carried out for 2 hours during which the test tube containing each material moved through 10 cups to measure the change in pH of the materials at the periods of 1,5,15 minutes and so on. Immediately after removal of the test tube, the pH of the saline was measured using a pH meter. After 2 hours of mixing the test tube, containing the cement, it was removed from the last cup (the tenth cup) and immersed in another cup of saline for 22 hours to measure the pH after 24 hours from the start of mixing.

-Testing the strength properties:

Customized hollow Teflon cylinders of 3.1 mm diameter and 6.2 mm depth were used for the production of compressive strength specimens.

The cylinders were placed on a small glass slab and filled with mixed cements. Each cylinder was filled with the mixed material using a spatula, and then covered with another glass slab.

After 24 hours from the beginning of mixing, the glass slabs were slid off, and the specimens were pushed out gently to be tested after 24 hours and 21 days. All the specimens were tested on the testing machine to determine the compressive and tensile strengths.

-Compressive strength:

The long specimens (6.2mm depth) were tested for the compressive strength. Each specimen was individually vertically mounted on a computer controlled materials testing machine (Model LRX-plus; Lloyd Instruments Ltd., Fareham, UK) with a load cell of 5 kN and data were recorded using the computer software (Nexygen-MT; Lloyd Instruments). Then the samples were statically loaded (in compression manner) using a stainless-steel rod ended with flat plate (40mm x 60mm) attached to the upper movable compartment of the machine at a crosshead speed of 0.5 mm/min until failure.

**Figure 1:**
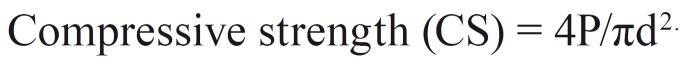
Equation.

The maximum failure load was recorded in N and converted into MPa. The compressive strength which was calculated from the recorded peak load was divided by sample surface according to the following equation; (Fig. 1).

Where *P* is the load (N) at the fracture point and d is the diameter (mm) of the cylindrical specimen.

-Diametral tensile strength

The short specimens (3.1mm depth) were tested for the diametral tensile strength. Each specimen was individually mounted on a computer controlled materials testing machine (Model LRX-plus; Lloyd Instruments Ltd., Fareham, UK) with a load cell of 5 kN and data were recorded using computer software (Nexygen-MT; Lloyd Instruments). Then the samples were statically loaded diametrically using a stainless-steel rod with flat end (10cm width 5cm breadth) attached to the upper movable compartment of the machine at a crosshead speed of 0.5 mm/min until failure.

**Figure 2:**
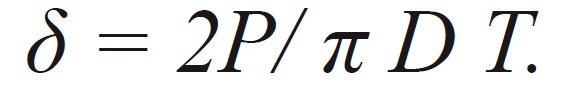
Equation.

The maximum failure load was recorded in N and converted into MPa. The diametral compressive strength was calculated from the following equation; (Fig. 2).

Where δ = diametral tensile strength (MPa), *P* = load at failure (N), π = 3.14, D = disc diameter (mm) and T = disc thickness (mm).

-Testing the adhesive bond strength:

•Tooth specimens

Recently extracted human maxillary anterior teeth were used for testing the adhesive bond strength. Roots of all the teeth were cut off with a water-cooled fine carborundum disc. 2.0 mm-thick slices were obtained from the cervical, middle, and apical root thirds. The cervical sections were cut 1 mm apical to the cementoenamel junction, the middle sections were cut 5 mm apical to the cementoenamel junction, and the apical sections were cut 8 mm apical to the cementoenamel junction. Eight slices were used for each tested cement.

The diameter of the root canal slices was standardized as follow; each slice was enlarged by means of a conical steel bur mounted in a low-speed hand piece. During specimen preparation, the root canals were irrigated with distilled water. Next, the specimens were immersed in a 2.5% sodium hypochlorite solution for 15 minutes, dried and then immersed in a 17% EDTA solution for 3 minutes. Finally washed in distilled water to remove the smear layer.

•Push out bond strength

**Figure 3:**
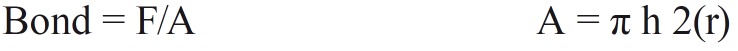
Formula.

Each cement was mixed according to the predetermined powder/water ratio. After mounting in a loading fixture, each sample was subject to compressive loading via the computer controlled materials testing machine (Model LRX-plus; Lloyd Instruments Ltd., Fareham, UK) with a load cell of 5kN. Data were recorded using the computer software (Nexygen-MT; Lloyd Instruments) and loaded at a crosshead speed of 0.5 mm/min. Load applied by plungers of diameter size (1 mm). The selected diameter of the plunger was positioned so that it only contacts the filling to displace it downward. The maximum failure load was recorded in N and converted into MPa. The bond strength was calculated from the recorded peak load divided by the computed surface area ,as calculated by the following formula; (Fig. 3).

Where π is the constant 3.14, r1 radius and h is the thickness of the sample in millimeters.

Failure was manifested by extrusion of the filling material and confirmed by sudden drop along load-deflection curve recorded by computer software.

-Statistical analysis:

Data were presented as means and standard deviation values. One-Way ANOVA was used to compare the different tested groups. Duncan’s post-hoc test was used when ANOVA was significant for pairwise comparison between tested variables. Statisical analysis was performed with IBM® SPSS® 20 (SPSS, Inc., IBM Corporation, NY, USA).

## Results

Means and standard deviations (SD) of setting times (Min), compressive strength (MPa) after 24 hours and 21 days, diametral tensile strength (MPa) after 24 hours and 21 days and Push out Bond strength (MPa) for the different materials are presented in  Table 1.

**Table 1:**
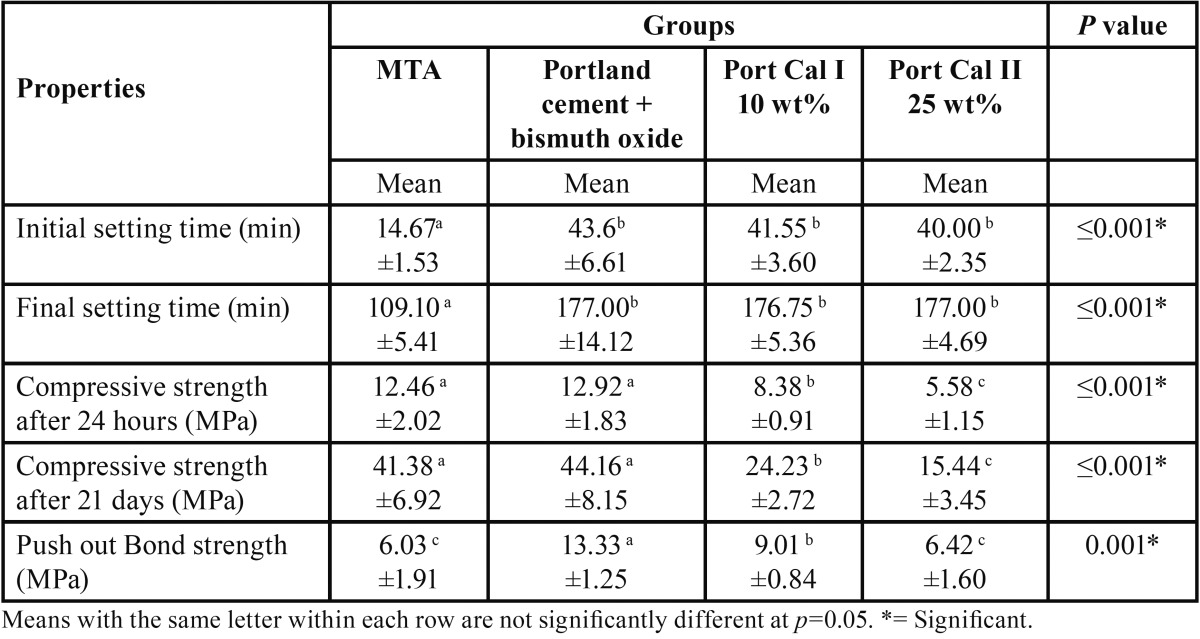
Means and standard deviations (SD) of the setting time (min), compressive strength (MPa), diametral tensile strength (MPa), push out bond strength (MPa) for the different materials.

-Mean setting time (Min):

•Initial setting time

Means and standard deviations (SD) of initial setting time (Min) for the different materials are presented in  Table 1.

MTA (14.67±1.53 min) showed the lowest statistical mean Initial setting time (Min) compared to Portland cement + bismuth (43.60±6.61 min), Port Cal I 10 wt% (41.55±3.60 min) and Port Cal II 25 wt% (40±2.35 min) with only a highly significant difference between MTA and all other tested materials at *P* ≤ 0.001. No significant difference was recorded between other materials.

•Final setting time

Means and standard deviations (SD) of final setting time (Min) for the different materials are presented in  Table 1.

MTA (109.10±5.41 min) showed the lowest statistical mean final setting time (Min) compared to Portland cement + bismuth (177±14.12 min), Port Cal I 10 wt% (176.75±5.36 min) and Port Cal II 25 wt% (177±4.69 min) with only a highly significant difference between MTA and all other tested materials at *P* ≤0.001. No significant difference was recorded between the other materials.

-Compressive strength (MPa):

•After 24 hours

MTA (12.46±2.02 MPa) and Portland cement + bismuth (12.92±1.83 MPa) showed the highest statistical mean compressive strength (MPa) followed by Port Cal I 10 wt% (8.38±0.91 MPa) and Port Cal II 25 wt% (5.58±1.15 MPa) at *P* ≤0.001. Compressive strength of Port Cal I was significantly higher than that of Port Cal II (*P* ≤0.005).

•After 21 Days

MTA (41.38±6.92 MPa) and Portland cement + bismuth oxide (44.16±8.15 MPa) showed the highest statistical mean compressive strength (MPa) followed by Port Cal I 10 wt% (24.23±2.72 MPa) and Port Cal II 25 wt% (15.44±3.45 MPa) at *P* ≤0.001. Compressive strength of Port Cal I was significantly higher than that of Port Cal II.

-Push out Bond strength (MPa):

Portland cement + bismuth (13.33±1.25 MPa) showed the highest statistical mean push out bond strength (MPa) followed by Port Cal I 10 wt% (9.01±1.25 MPa) followed by MTA (6.03±1.91 MPa) and Port Cal II 25 wt% (6.42±1.6 MPa) which showed no significant difference in mean Push out Bond strength (MPa) at *P* =0.005.

-pH values of different material:

 Table 2 shows the pH values of the different materials and their average at different times.

**Table 2:**
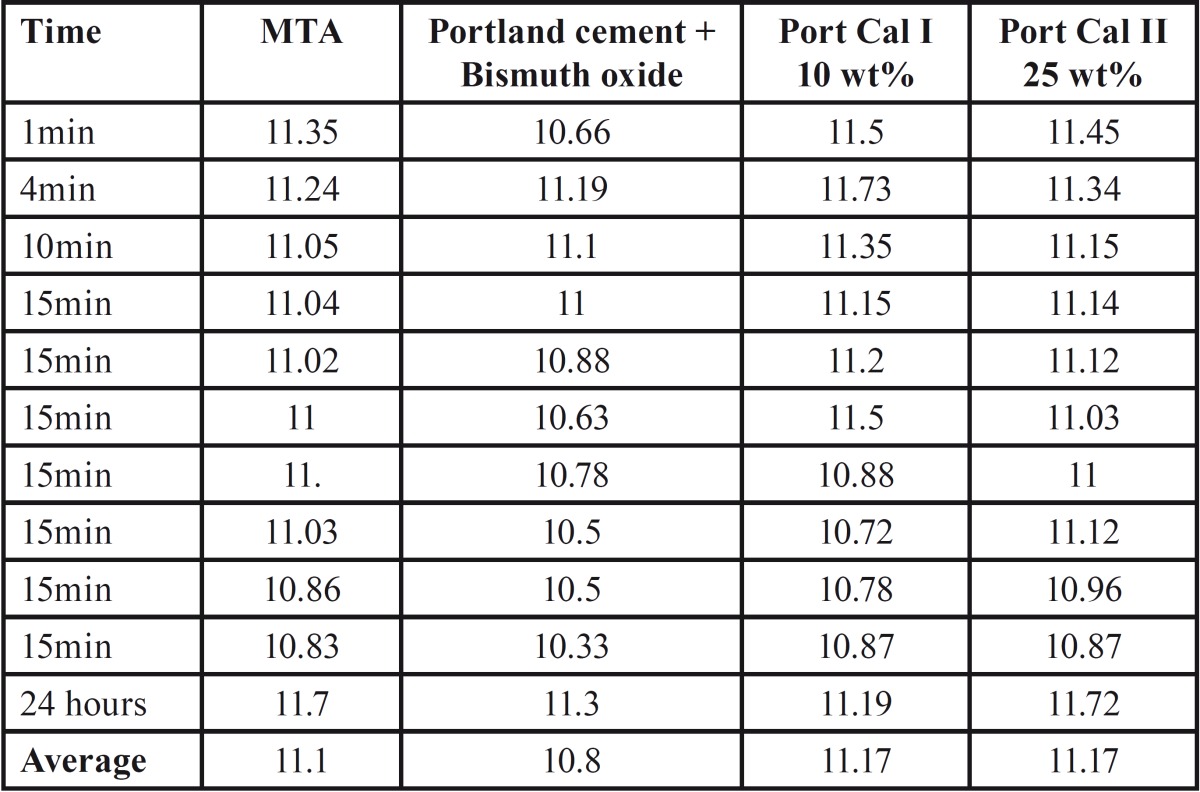
pH values of different materials and their average at different times.

## Discussion

Recently, the possibility of using Portland cement (PC) as a cheap alternative to MTA attracts the attention of researchers. However, it might be a logical step to try to introduce some chemical changes in constituents of Portland cement in order to evaluate the effect of these changes on the biological and physical properties of the material. Therefore the present part of the study was planned to develop and evaluate the physical properties of a new pulp capping material developed from Portland cement in order to test it biologically in the second part of the study.

The addition of 20% bismuth oxide was done for its radiopacifying effect in order to simulate MTA according to a study by Bueno *et al.* (14).

Calcium hydroxide was added to Portland cement in an attempt to add antimicrobial effect to its biological characteristics. Also its mineralization effect which can be attributed to the hydroxyl ions released that induce an alkaline pH. This pH induces liquefaction necrosis in the superficial portion of the pulp, deeper portions of the pulp witness neutralization so stimulates hard tissue formation.

In the present study MTA showed the lowest statistical mean initial and final setting times compared to Portland cement+bismuth oxide, Port Cal I and Port Cal II, with no significant difference recorded between the other materials. These findings might be attributed to the addition of exogenous particles that interfered with the hydration mechanism of Portland cement, delaying matrix formation, and consequently increasing the setting time of the cement. However, Anand *et al.* (15) recorded that the addition of a substance containing calcium to Portland cement will reduce its setting time as it reduces the incorporation of water and allow the cement to resist hydrostatic pressure at early stages avoiding its leaching out.

Both Portland cement and MTA are rich in calcium ions, which are converted to calcium hydroxide upon contact with water. Calcium hydroxide dissociates into calcium and hydroxyl ions, which increases the pH of the solution. Thus the variation in the concentration of calcium hydroxide leads to different pH values (16). The percentages of calcium hydroxide added to Portland cement did not seem to be enough to create a significant difference in the pH. This finding came in agreement with Gonçalves *et al.* (13).

In this study, pH values ranged from 10-11.7, however Torabinejad *et al.* (17) reported values higher than 12 for MTA. This difference in pH measurement could be due to pH was directly measured by Torabinejad *et al.* from the cement mass using electrodes rather than immersion of the samples in deionized distilled water.

The significance of measuring the compressive strength is to test the ability of the experimental materials to withstand compressive forces of condensation of the overlying restorative materials. MTA and Portland cement+bismuth oxide showed the highest statistical mean compressive strength followed by Port Cal I that was significantly higher than Port Cal II at both periods. These findings came in agreement with Camilleri (18) who found that Portland cement and MTA had comparable compressive strength. But in another study, the authors found that MTA has higher compressive strength due to the fineness of its particles that influences the rate of hydration (19).

In the present study, Portland cement+bismuth oxide had the highest mean push out bond strength followed by Port Cal I, MTA and Port Cal II. These findings came in agreement with the manufacturer and Iacono *et al.* (20) who claimed that Endocem MTA has lower bond strength initially. However, the bond strength increases over time to be comparable. On the other hand, these findings disagreed with those reported by Amoroso-Silva *et al.* (21) who found that push out bond strength of MTA and Portland cement was similar due to their similar chemical composition.

In conclusion, the strength properties and push out bond strength of Portland cement were adversely affected by addition of calcium hydroxide especially with a ratio of 25 wt%, however, the setting time and pH were not affected. Taking the setting time, push out bond strength and pH value into account, addition of 10 wt% calcium hydroxide to Portland cement produces a new pulp capping material with acceptable physical and adhesive properties.
